# Critical slowing down near a magnetic quantum phase transition with fermionic breakdown

**DOI:** 10.1038/s41567-023-02156-7

**Published:** 2023-07-31

**Authors:** Chia-Jung Yang, Kristin Kliemt, Cornelius Krellner, Johann Kroha, Manfred Fiebig, Shovon Pal

**Affiliations:** 1 Department of Materials, ETH Zurich, Zurich, Switzerland; 2 Physikalisches Institut, Goethe-Universität Frankfurt, Frankfurt, Germany; 3 Physikalisches Institut and Bethe Center for Theoretical Physics, Universität Bonn, Bonn, Germany; 4 School of Physical Sciences, National Institute of Science Education and Research, HBNI, Jatni, India

**Keywords:** Phase transitions and critical phenomena, Electronic structure, Terahertz optics

## Abstract

When a system close to a continuous phase transition is subjected to perturbations, it takes an exceptionally long time to return to equilibrium. This critical slowing down is observed universally in the dynamics of bosonic excitations, such as order-parameter collective modes, but it is not generally expected to occur for fermionic excitations. Here using terahertz time-domain spectroscopy, we find evidence for fermionic critical slowing down in YbRh_2_Si_2_ close to a quantum phase transition between an antiferromagnetic phase and a heavy Fermi liquid. In the latter phase, the relevant quasiparticles are a quantum superposition of itinerant and localized electronic states with a strongly enhanced effective mass. As the temperature is lowered on the heavy-Fermi-liquid side of the transition, the heavy-fermion spectral weight builds up until the Kondo temperature *T*_K_ ≈ 25 K, then decays towards the quantum phase transition and is, thereafter, followed by a logarithmic rise of the quasiparticle excitation rate below 10 K. A two-band heavy-Fermi-liquid theory shows that this is indicative of the fermionic critical slowing down associated with heavy-fermion breakdown near the quantum phase transition. The critical exponent of this breakdown could be used to classify this system among a wider family of fermionic quantum phase transitions that is yet to be fully explored.

## Abstract (web summary)

YbRh_2_Si_2_ has a quantum phase transition between an antiferromagnetic phase and a so-called heavy-Fermi-liquid state. Measurements of critical slowing down suggest that the heavy-fermion quasiparticles break down at the transition.

## Main

**Fig. 1:**
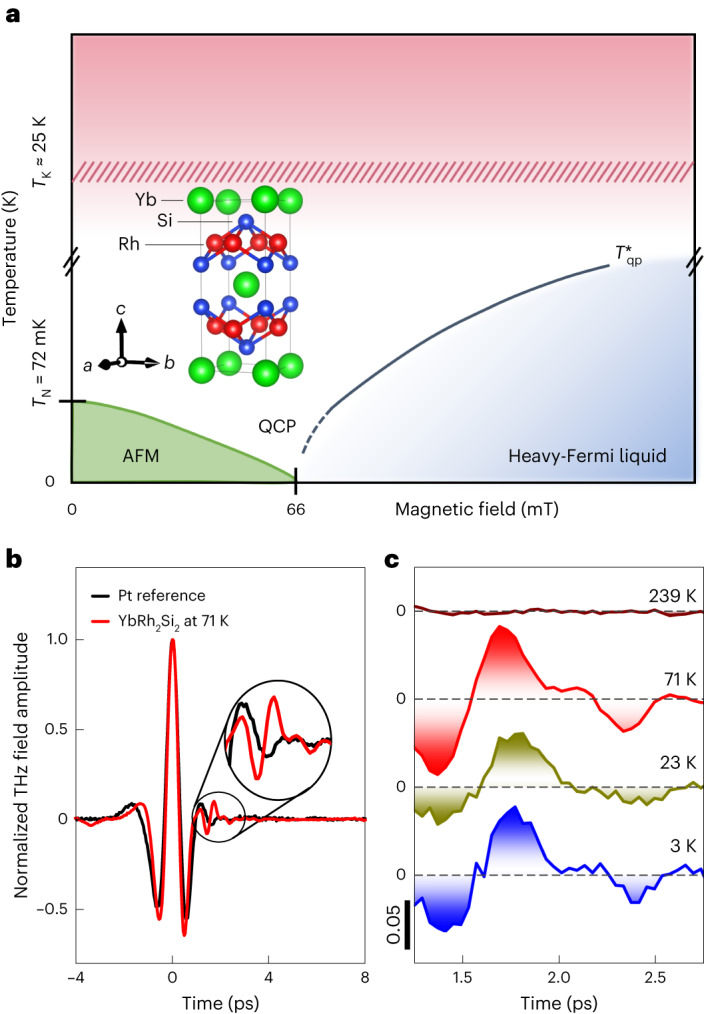
Exploring fermionic quantum criticality by time-resolved THz reflectivity. **a**, Schematic phase diagram of YbRh_2_Si_2_ with characteristic energy scales as defined in the text. AFM, antiferromagnetic phase. **b**, Reflected THz signal from the YbRh_2_Si_2_ sample (red) and from a Pt mirror reference (black). The time traces are normalized by the maximum field amplitude at *t* = 0 ps. The delayed, purely Kondo-related ‘echo’ pulse is visible in the interval between 1.3 and 2.6 ps, where it distinctly differs from the Pt reference signal. **c**, Subtracted signal of the delayed pulse in YbRh_2_Si_2_ at *B*_⊥_ = 70 mT for various temperatures. Source data

At a continuous phase transition, the ordered and the disordered phases have the same energy. As a consequence, the fluctuations between these two states become infinitely slow. This so-called critical slowing down (CSD) is universally observed in the dynamics of classical fields that are bosonic in nature but vanishes at the phase transition, like the magnetization associated with bosonic magnons, in the case of ferromagnetic order^1^. In contrast, the CSD of fermionic excitations or quasiparticles is generally not expected to occur since fermions, as elementary particles, are thought to be indestructible. However, certain quantum materials known as heavy-fermion (HF) compounds host composite fermionic quasiparticles. These are quantum superpositions of itinerant and localized (that is, heavy) electron states generated by the Kondo effect^2,3^ and have low binding energy parameterized by the Kondo energy scale, the lattice Kondo temperature *T*_K_. At a quantum phase transition (QPT) in such materials (Fig. 1a), these brittle, heavy quasiparticles are assumed to disintegrate^4–6^ despite their fermionic nature. Defining their spectral weight, that is, the probability of their existence, as the order parameter of such a fermionic QPT, one may expect the CSD of the fermionic quasiparticle oscillations—a unique signature of critical HF quasiparticle destruction, as opposed to bosonic order-parameter fluctuations.

Using time-resolved terahertz (THz) spectroscopy, we directly observe such fermionic CSD as a suppression of the heavy-particle hybridization gap and a flattening of the associated band. This ‘softening’ expands the region in momentum space where resonant THz absorption is allowed. We observe this as an increase instead of a Kondo-weight-loss-generated decrease of the Kondo-related THz signal towards the quantum-critical point (QCP), before the heavy quasiparticle band vanishes altogether below a breakdown temperature ${T}_{{{{\rm{qp}}}}}^{* }$ (ref. ^7^). Moreover, we identify a critical exponent in this behaviour, which may, thus, lead to the classification of fermionic quantum criticality in analogy to the criticality of thermodynamic phase transitions.

Signatures of Kondo quasiparticle destruction were suspected to have been seen in the dynamical scaling of the magnetic susceptibility^8^, specific heat measurements^9^, Hall effect measurements of the carrier density^10^ and optical conductivity measurements^11^. The conjectures required for interpreting these measurements have been challenged, however^12,13^. Time-resolved THz spectroscopy is a unique tool for probing heavy quasiparticle dynamics and for resolving these questions. Specifically, HF materials respond to an incident ultrashort THz pulse by emitting a time-delayed reflex^14^. This ‘echo’ is a response from the reconstructing Kondo ground state after its destruction by the incident pulse. Hence, time acts as a filter separating the Kondo-sensitive delayed pulse from the Kondo-insensitive main pulse so that the former is a background-free response of the HF state. Specifically, the amplitude and the delay time of the echo pulse are proportional to the HF spectral weight and the Kondo coherence time *τ*_K_ = 2πℏ/*k*_B_*T*_K_, respectively (with ℏ the reduced Planck constant and *k*_B_ the Boltzmann constant). This technique was introduced in experiments on CeCu_6−*x*_Au_*x*_ (refs. ^14–16^).

We now apply this new method to measure the fermionic CSD of YbRh_2_Si_2_ directly. YbRh_2_Si_2_ is a prototypical HF compound. In zero magnetic field, it is antiferromagnetic below the Néel temperature *T*_N_ = 72 mK. It undergoes a QPT to a Kondo HF liquid at a critical magnetic field of ${B}_{\perp }^{{{{\rm{cr}}}}}\approx 66$ mT perpendicular to the *c*axis^17,18^ (Fig. 1a) induced by the Ruderman–Kittel–Kasuya–Yosida (RKKY) magnetic interaction between the Yb moments^19–22^. Alternatively, a 6% substitution of Rh by Ir creates a QCP at zero field^23,24^. YbRh_2_Si_2_ has a Kondo temperature of *T*_K_ ≈ 25 K, which is high enough to enable a wide quantum-critical region and to permit us to search for signs of CSD in the range ${T}_{{{{\rm{qp}}}}}^{* } < T < {T}_{{{{\rm{K}}}}}$, in contrast to CeCu_6−*x*_Au_*x*_. In our temperature-dependent, time-resolved THz reflection spectroscopy measurements, we cross the QCP by varying the magnetic field or the Ir concentration. The 1.5-cycle THz pulses of approximately 2 ps duration are incident onto the *c*-cut Yb(Rh_1−*x*_Ir_*x*_)_2_Si_2_ samples (*x* = 0, 0.06). The echo pulses are analysed as described elsewhere^14^. With *T*_K_ = 25 K, we obtain a delay time *τ*_K_ ≈ 1.9 ps for these in agreement with the data in Fig. 1b,c. We, therefore, choose the time window for the analysis as being from 1.3 to 2.6 ps (see Supplementary Fig. 2 for the verification of the robustness of our results with respect to variations of this time window).

**Fig. 2:**
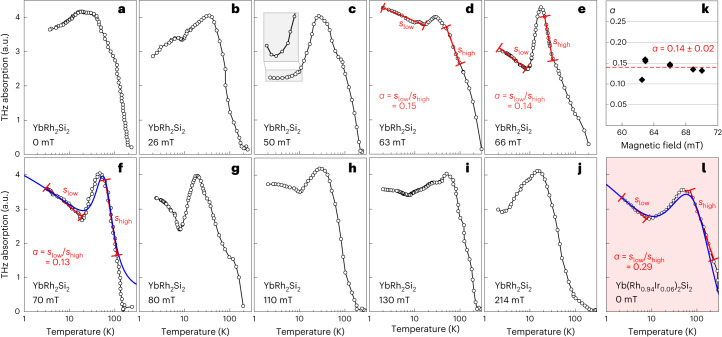
Temperature dependence of the resonant THz absorption across the QCP in YbRh_2_Si_2_. **a**–**j**, Evolution of the THz absorption by resonant Kondo quasiparticle excitations for magnetic fields *B*_⊥_ as indicated. The weights are derived from the integrated intensity of the echo pulses emitted in the time window 1.3–2.6 ps (Fig. 1b,c). **k**, Fermionic critical exponent *α* as extracted for various magnetic fields *B*_⊥_ near the QPT. **l**, Temperature dependence of the resonant THz absorption in quantum-critical Yb(Rh_1.94_Ir_0.06_)_2_Si_2_. Fits of equation (1) are plotted as blue lines in **f** and **l**. The absolute values of the data may shift by 1 to 10%, depending on the experimental conditions, without, however, affecting the value of *α*. a.u., arbitrary units. Source data

Figure 2 shows the time-integrated intensity of the THz echo pulse for Yb(Rh_1−*x*_Ir_*x*_)_2_Si_2_ for *x* = 0 at various values of *B*_⊥_ across the QPT, as well as for *x* = 0.06 at *B*_⊥_ = 0. All plots exhibit the Kondo-like logarithmic increase of the spectral weight down from high temperatures, reaching a maximum in the region around the crossover temperature of *T*_K_ = 25 K. The kink near 100 K visible in all plots is caused by the population of the first crystal-electric-field excitation^25,26^. Below the peak temperature, the signal initially decreases with temperature for all magnetic fields. This can be attributed to the reduced thermal broadening of THz-induced interband transitions and is reproduced by the theory introduced below.

On the antiferromagnetic side of the QCP (Fig. 2a,b), the signal continues to decrease but remains finite down to the lowest experimentally achieved temperature of 2 K. Note that this temperature range (2.0 K ≤ *T* ≤ 20 K) and field range (*B*_⊥_ ≲ 66 mT) are both within the white area of the phase diagram in Fig. 1a, the so-called quantum-critical fan^9,24^, where the thermodynamic and transport properties are dominated by quantum-critical fluctuations^11,17,18^.

Our THz time-delay spectroscopy is not directly sensitive to these fluctuations but exclusively to the HF quasiparticle spectral weight^14,15^. Therefore, the behaviour in Fig. 2a,b indicates that the Kondo effect remains partially intact in this temperature range. This is reasonable since we are still a factor of approximately 25 above *T*_N_ so that the heavy quasiparticles are not entirely destroyed by the impending antiferromagnetic order.

Near quantum criticality (Fig. 2d–f,l), the temperature dependence changes drastically. The initial signal decrease with temperature is now followed by a logarithmic increase of the THz echo signal, which persists down to the lowest observed temperature. Note the qualitative similarity between the field-tuned (Fig. 2e) and chemically tuned (Fig. 2l) quantum-critical systems, albeit with different logarithmic slopes. On the HF-liquid side of the QCP, the signal increase towards the lowest temperatures is still present and gradually fades away with distance from the QCP (Fig. 2g–j). Its onset may also be conjectured at 50 mT on the antiferromagnetic side (see the inset in Fig. 2c).

**Fig. 3:**
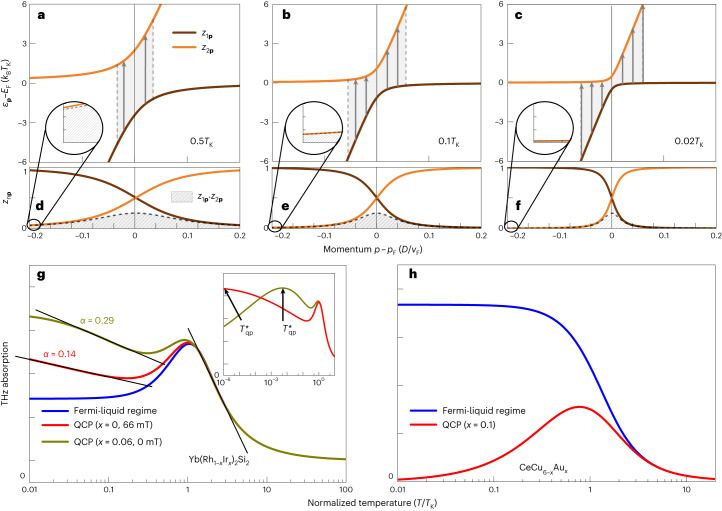
Band structure and Kondo weight calculations towards the QCP. **a**–**c**, Band structure of the conduction band (steep slope) and the Kondo state (flat band) resulting in a hybridized lower (brown) and upper (orange) branch, for temperatures *T* = 0.5*T*_K_, 0.1*T*_K_, 0.02*T*_K_, respectively, as indicated. **d**–**f**, Corresponding momentum and temperature-dependent quasiparticle weights ${z}_{1\mathbf{p}}$, ${z}_{2\mathbf{p}}$ in the lower (brown) and upper (orange) bands (see equation (5)) as well as the product ${z}_{1\mathbf{p}}\cdot {z}_{2\mathbf{p}}$, all calculated from the two-band critical-Fermi-liquid theory. **g**,**h**, Resonant THz absorption strength at (red and dark yellow) and away from (blue) the QCP as calculated for the system parameters of Yb(Rh_1−*x*_Ir_*x*_)_2_Si_2_, (*x* = 0, 0.06) (**g**) and CeCu_6−*x*_Au_*x*_(**h**). In quantum-critical CeCu_5.9_Au_0.1_, the low value of *T*_K_ ≈ 8 K renders the temperature window ${T}_{{{{\rm{qp}}}}}^{* } < T < {T}_{{{{\rm{K}}}}}$ too narrow for logarithmic behaviour to be observed. *p*_F_ and *v*_F_ denote the Fermi momentum and Fermi velocity, respectively. See the text for more details. Source data

To understand the striking logarithmic increase towards low temperature, we analyse the THz echo signal theoretically. We summarize this analysis here and elaborate on its technical aspects in Methods. In a HF system, the strongly correlated, flat band produced by the Kondo effect hybridizes with the light conduction band to generate a structure with a lower (*n* = 1) and an upper (*n* = 2) band with an avoided crossing^15,27,28^, as shown in Fig. 3a–c. Low-temperature thermodynamic and transport experiments probe the lower, occupied band only, whereas resonant THz spectroscopy covers transitions between both bands, requiring a two-band theory. Our critical-HF-liquid theory shows (Methods) that the *n* = 1, 2 bands with dispersions ${\varepsilon }_{n\mathbf{p}}$ (where $\mathbf{p}$ is the crystal-electron momentum) have distinct momentum- and temperature-dependent spectral weights ${z}_{n\mathbf{p}}$. This is a crossover from ${z}_{n\mathbf{p}}\approx 1$ in the strongly dispersive region to ${z}_{n\mathbf{p}}=a\,{z}_{0}\ll 1$ in the flat region of these bands (Fig. 3d–f and insets). Here, *a* ≪ 1 is the spectral weight of the local, single-ion Kondo resonance, which builds up logarithmically from above *T*_K_ and then saturates towards a constant value for *T* < *T*_K_. Further, ${z}_{0}={(T/{T}_{0})}^{\alpha }$ is a suppression factor with a critical exponent *α*. It describes the destruction of the quasiparticle spectral weight as the QCP is approached on lowering the temperature *T* below the onset temperature for quantum criticality, *T*_0_. For the latter, experiments revealed that *T*_0_ ≈ *T*_K_ in YbRh_2_Si_2_ (ref. ^29^).

As mentioned, the THz echo pulse at *τ*_K_ = 2πℏ/*k*_B_*T*_K_ is solely sensitive to the breakup-and-recovery dynamics of the HFs and not other THz absorption channels^14–16^. Therefore, its intensity exclusively depends on the quasiparticle weight and the phase space available for THz-induced excitations. Specifically, the echo-pulse intensity is proportional to the probability:

1 $$P(T)=A\int\mathrm{d}^{3}p\,{z}_{1\mathbf{p}}\,{z}_{2\mathbf{p}}\,f({\varepsilon }_{1\mathbf{p}})\,[1-f({\varepsilon }_{2\mathbf{p}})]\,W({{\Delta }}{\varepsilon }_{\mathbf{p}})$$

for the resonant excitation of electrons from the lower to the upper band at an energy difference ${\Delta}{\varepsilon }_{\mathbf{p}}={\varepsilon }_{2\mathbf{p}}-{\varepsilon }_{1\mathbf{p}}$. Here, $f({\varepsilon }_{n\mathbf{p}})$ is the Fermi–Dirac distribution function. In equation (1), it describes the probability that the *n* = 1 band is occupied and that the *n* = 2 band is empty before the THz absorption process. *W*(ℏ*ω*) is the spectrum of the incident THz pulse, which is a Gaussian distribution of width *Γ* centred around the central frequency *Ω*_THz_. With ${{\Delta }}{\varepsilon }_{\mathbf{p}}\approx \hslash {{{\varOmega }}}_{{{{\rm{THz}}}}}$, the THz-induced interband transition becomes resonantly allowed. The integral runs over all electron momenta, and the factor *A* is a temperature-independent constant, proportional to the intensity of the incident THz pulse and to the modulus squared of the electric–dipole transition-matrix element between the two bands.

When the probability for HF formation, $a\,{z}_{0}\propto {(T/{T}_{{{{\rm{K}}}}})}^{\alpha }$, tends to zero at the QCP, the heavy bands flatten according to the two-band HF-liquid theory. Also, the hybridization gap vanishes, and with that the quasiparticle energy in the heavy regions of both bands (*n* = 1, 2) approaches the Fermi energy *E*_F_ (Fig. 3d–f). This means that the oscillation frequency of fermionic quasiparticles, ${\omega }_{n\mathbf{p}}=({\varepsilon }_{n\mathbf{p}}-{E}_{{{{\rm{F}}}}})/\hslash$, vanishes, which is indicative of a fermionic CSD. In turn, it implies an expansion of the region in the momentum space where resonant THz transitions are allowed, seen as a broadening of the shaded areas in Fig. 3a–c. The interplay of these two counteracting effects, namely quasiparticle destruction and phase-space expansion, leads to a non-monotonic temperature dependence of the THz absorption strength *P*(*T*). In equation (1), *P*(*T*) depends on the bare quasiparticle weight *z*_0_(*T*) via ${\varepsilon }_{n\mathbf{p}}$, ${z}_{n\mathbf{p}}$, *n* = 1, 2, and ${{\Delta }}{\varepsilon }_{\mathbf{p}}$; see equations (4) and (5). A careful expansion of all these dependencies for small *z*_0_(*T*) and performing the integration in equation (1) predicts a logarithmic increase of $P(T)\propto \ln [1/{z}_{0}(T)]$ towards low temperatures down to the region of ${T}_{{{{\rm{qp}}}}}^{* }$. The full numerical evaluation of equation (1) leads to the behaviour shown in Fig. 3g. This reproduces the Kondo maximum near *T*_K_ ≈ 25 K, and at quantum criticality, it indeed shows a logarithmic increase within an intermediate temperature window ${T}_{{{{\rm{qp}}}}}^{* } < T < {T}_{{{{\rm{K}}}}}$ according to

2 $$P(T)=\alpha \,A\,\ln ({T}_{{{{\rm{K}}}}}/T).$$

Observing this behaviour in Fig. 2d–g,l is, thus, a unique experimental signature of fermionic quasiparticle CSD in the Yb(Rh_1−*x*_Ir_*x*_)_2_Si_2_ system.

Upon further decreasing the temperature to $T < {T}_{{{{\rm{qp}}}}}^{* }$, *P*(*T*) approaches zero as the HF weight disappears altogether (inset of Fig. 3g). The low-temperature scale ${T}_{{{{\rm{qp}}}}}^{* }$ is, thus, defined as the position of the signal maximum between the logarithmic increase and its ultimate collapse towards *T* → 0. As seen in Fig. 3g (inset), ${T}_{{{{\rm{qp}}}}}^{* }$ depends on the critical exponent *α* and is several orders of magnitude lower than the Kondo scale of approximately *T*_K_ and therefore possibly undetectably small. A low-temperature scale T* has also been observed as a maximum in the magnetic susceptibility^24^ whose microscopic origin, however, remains unclear. Since in Yb(Rh_1−*x*_Ir_*x*_)_2_Si_2_, T* and our theoretically predicted ${T}_{{{{\rm{qp}}}}}^{* }$ are in the same temperature range, we conjecture that both may have the same physical origin, namely the competition between fermionic CSD and quasiparticle breakdown at the QCP. As a crossover temperature, ${T}_{{{{\rm{qp}}}}}^{* }$ remains non-zero, but can be exceedingly small, depending on *α* (inset of Fig. 3g). Note that away from criticality (blue curve, Fig. 3g), quasiparticles persist (${z}_{0}(T)={{{\rm{const.}}}}$), so that the logarithmic low-temperature behaviour does not occur, in agreement with Fig. 2h–j. The blue curves in Fig. 2f,l represent the evaluation of equation (1) for the spectrum *W*(ℏ*ω*) of the THz pulses used in our experiment. Considering that *α* is the only adjustable parameter apart from the overall signal amplitude and that we use the same value *T*_K_ ≈ 25 K for both curves, the agreement between theory and data is excellent.

We can now extract the critical exponent *α* by comparing the logarithmic slope *s*_low_ associated with the CSD at low temperatures (${T}_{{{{\rm{qp}}}}}^{* } < T < {T}_{{{{\rm{K}}}}}$) from equation (2) with the slope *s*_high_ of the standard logarithmic behaviour of the Kondo weight at high temperature (*T* > *T*_K_) according to $P(T)=A\,\ln ({T}_{{{{\rm{K}}}}}/T)$. Specifically, *s*_high_ is extracted from the temperature window between the signal maximum and the crystal-electric-field kink near 100 K. This directly leads to *α* = *s*_low_/*s*_high_. We find from the experimental data that *α* = 0.14 ± 0.02 for Yb(Rh_1−*x*_Ir_*x*_)_2_Si_2_ in the critical region *x* = 0 and 63 mT ≤ *B*_⊥_ ≤70 mT in Fig. 2d–f and that *α* = 0.29 at *x* = 0.06 and *B*_⊥_ = 0 in Fig. 2l. Such different critical behaviours for magnetic-field and chemical-pressure tuning reflects that, for different tuning parameters, the QCPs are of a different nature, which has also been observed in response functions^30^ quantifying the critical behaviour of bosonic fields.

Our theory also explains why the logarithmic low-temperature increase of THz absorption indicating CSD cannot be observed in the CeCu_6−*x*_Au_*x*_ system. For this material, the ratio ${T}_{{{{\rm{K}}}}}/{T}_{{{{\rm{qp}}}}}^{* }$ is substantially smaller than in the Yb(Rh_1−*x*_Ir_*x*_)_2_Si_2_ system, so that the effects of the buildup of the Kondo weight and of the CSD overlap to such an extent that the latter is obscured, as seen in Fig. 3h and in agreement with experiment^14^.

**Fig. 4:**
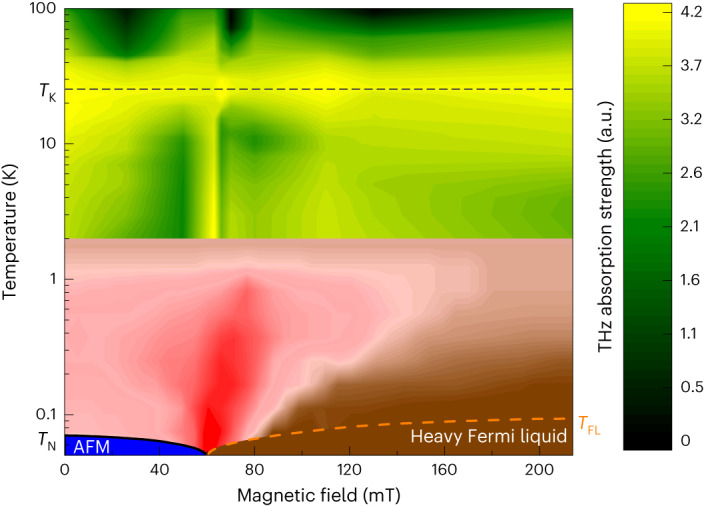
Phase diagram of field-tuned YbRh_**2**_Si_**2**_. The yellow-green area is where resonant THz absorption occurs, as measured by THz time-delay spectroscopy, using the discrete magnetic-field values shown in Fig. 2. The data are corrected for experimental variations of the amplitude *A* from equation (1). The red-brown area is the low-temperature phase diagram inferred from a convolution of magnetotransport, magnetostriction and magnetization measurements^31^. Our spectroscopic results are consistent with the low-temperature phase diagram and extend it to high temperatures, showing non-Fermi liquid behaviour up to approximately 25 K, as expected. The critical breakdown of heavy quasiparticles is seen on the HF side of the QPT (bright yellow region for *B* > 66 mT), whereas no heavy quasiparticles are present on the antiferromagnetic side (dark green region for *B* < 66 mT). Figure adapted with permission from ref. ^31^ under a Creative Commons license CC BY. Source data

Our findings are summarized in the *T* versus *B*_⊥_ phase diagram of Fig. 4. On the HF-liquid side of the QCP ($B > {B}_{\perp c}^{{{{\rm{cr}}}}}=66$ mT), *P*(*T*) is logarithmically enhanced due to the CSD effect, as explained by our two-band HF-liquid theory, signalling the critical behaviour of the HF quasiparticles up to *T* ≲ 10 K. In contrast, on the antiferromagnetic side (*B*_⊥_ ≲ 50 mT), we observe a reduction of the Kondo-weight-related absorption but no fermionic CSD. This reduction is different from thermodynamic and response measurements, which are dominated by quantum fluctuations. Our measurements are sensitive to the quasiparticle dynamics only. This suggests that in this region, the RKKY interaction^19–21^ strongly affects the quasiparticle dynamics such that the two-band HF-liquid theory is not valid here. Figure 4 shows how our measurements connect to the quantum-critical fan^31^ observed at the lowest temperatures (*T* < 2 K).

To conclude, we observed a logarithmic low-temperature increase in the resonant quasiparticle excitation probability *P*(*T*) near a magnetic QPT in HF materials. We identified this logarithmic increase as a unique signature of fermionic quasiparticle CSD, that is, a vanishing quasiparticle frequency near a QPT with fermionic breakdown. Since, in contrast to the thermodynamic and transport properties, our time-resolved THz spectroscopy is exclusively sensitive to the HF quasiparticle dynamics as opposed to thermal fluctuations, we could further extract the fermionic critical exponent *α* of the vanishing quasiparticle weight. The critical behaviour of *α* suggests that we can define the heavy quasiparticle weight as an order parameter for QPTs with fermionic breakdown. This work may lead to the classification of fermionic QPTs in terms of their critical exponent, analogous to thermodynamic phase transitions.

## Methods

### Experimental

Single-crystalline, *c*-oriented Yb(Rh_1−*x*_Ir_*x*_)_2_Si_2_ platelets (*x* = 0, 0.06) with dimensions of 2 × 3 × 0.07 mm^3^ were grown from an indium flux as described in the literature^23^. The sample surface was freshly polished before the THz measurements. Samples were mounted onto a Teflon holder. Two permanent magnets, placed above and below a sample, generated a magnetic field of up to 214 mT in the easy magnetic plane perpendicular to the tetragonal *c*axis. We used a temperature-controlled Janis SVT-400 helium-reservoir cryostat operable in the range from 1.9 to 325 K. For measurements with finer tuning of the magnetic field, we used Helmholtz coils mounted around the cryostat. The coils were made from a polyimide-coated Cu wire of thickness 1.8 mm with dimensions as follows: inner radius 8 cm, outer radius 13 cm and package thickness 7.5 cm. The coils provided a very large aperture for on-axis (14 cm) and off-axis (2–3 cm) experiments. By adding two soft-iron field guides sized 12 × 12 × 12 mm^3^, we generated magnetic fields of up to 180 mT, with a field homogeneity of approximately  1 % up to 2 cm from the centre, much larger than the sample dimensions. The THz experiments were performed in a 90^∘^ reflection geometry with light in the spectral range from 0.1 to 3 THz polarized perpendicular to the crystallographic *c*axis.

We generated single-cycle THz pulses by optical rectification in a 0.5-mm-thick (110)-cut ZnTe single crystal, using 90% of the amplified output of a Ti:sapphire laser (wavelength 800 nm, pulse duration 50 fs, pulse repetition rate 1 kHz and 2.5 mJ pulse energy). The energy of the THz pulse was a few nanojoules. The residual 10% of the 800-nm beam was then used for free-space electro-optic sampling of the reflected THz light from the sample. Both the THz and the 800-nm beams were collinearly focused onto a 0.5-mm-thick (110)-cut ZnTe detection crystal. To increase the accessible time delay between the THz and the 800-nm pulses, Fabry–Pérot resonances from the faces of the detection crystal were suppressed by a 2-mm-thick THz-inactive (100)-cut ZnTe crystal. The THz-inactive crystal was optically bonded to the back of the detection crystal. The THz-induced ellipticity of the 800-nm beam was measured using a quarter-wave plate, a Wollaston prism and a balanced photodiode.

### Theoretical

Ce- or Yb-based HF compounds can be described by the standard Anderson lattice model^3^, in which a single electron or hole in the 4*f* shell of each Ce or Yb atom, respectively, hybridizes with conduction electrons occupying a broad band of dispersion ${\varepsilon }_{\mathbf{p}}^{(0)}$ (measured relative to the Fermi energy *E*_F_). The temperature-dependent spectral distribution of this system can be computed, for example, by dynamical mean-field theory with the non-crossing approximation as the impurity solver^14^. The features of the resulting band structure at temperatures around and below *T*_K_ can be summarized as follows. The Kondo effect accumulates 4*f* spectral weight in resonance states at the Fermi level, which forms a weakly dispersive band of heavy Bloch states below the lattice coherence temperature. The parameters of these heavy states are controlled by the Kondo scale *T*_K_. Due to particle–hole asymmetry, their energy is shifted by *Δ* ≈ ±*k*_B_*T*_K_ above (particle-like HF systems) or below (hole-like HF systems) *E*_F_(refs. ^3,28^). The heavy-band states overlap with the conduction-electron states by an effective hybridization matrix element of order *V* ≈ *k*_B_*T*_K_, and their spectral weight *a*(*T*) is given by the spectral weight of the Kondo resonance. It is, therefore, strongly reduced with respect to unity, reaches *a*(0) = *T*_K_/*γ* ≪ 1 for *T* → 0, and decreases logarithmically for temperatures *T* > *T*_K_ (ref. ^3^). Here, *γ* is the effective energy broadening of the bare rare-earth 4*f* orbitals due to hybridization with the conduction-electron states. Denoting the quasiparticle frequency by *ω*, the two-band propagator for conduction and heavy electrons is thus

3 $${G}_{\mathbf{p}}(\omega )={\left(\begin{array}{ll}\omega -{\varepsilon }_{\mathbf{p}}^{(0)}&V\\ {V}^{* }&\frac{1}{a(T)}\left[\omega -{{\Delta }}-{{\varSigma }}(\omega )\right]\\ \end{array}\right)}^{-1}$$

We construct a phenomenological, critical Fermi liquid theory for this two-band system to describe the THz-induced, resonant transitions from the heavy to the light band. While the light conduction electrons can be assumed to be non-interacting, a residual interaction of the quasiparticles within the heavy band is taken into account, being described by the self-energy *Σ*(*ω*) in equation (3). This implies, by the expansion of *Σ*(*ω*) about the Fermi level, that there is an additional reduction of both the quasiparticle weight and of the heavy band shift by the local quasiparticle weight factor *z*_0_(*T*) = [1 − (∂*Σ*/∂*ω*)]^−1^, that is, *a* → *z*_0_*a* and *Δ* → *z*_0_*Δ*. It is this quasiparticle weight *z*_0_(*T*) that vanishes at the QCP in YbRh_2_Si_2_ due to critical quasiparticle destruction. We, thus, assume power law behaviour with a fermionic critical exponent *α* > 0 near the QCP, ${z}_{0}(T) \sim {(T/{T}_{{{{\rm{K}}}}})}^{\alpha }$ for *T* → 0 and *B*_⊥_ = *B*_⊥*c*_. The hybridized band structure is then calculated in a standard way by diagonalizing the matrix propagator (3), and the band dispersions are obtained as the poles of its eigenvalues:

4 $${\varepsilon }_{1,2\mathbf{p}}=\frac{1}{2}\left[{\varepsilon }_{\mathbf{p}}^{(0)}+{z}_{0}{{\Delta }}\pm \sqrt{{ \left({\varepsilon }_{\mathbf{p}}^{(0)}-{z}_{0}{{\Delta }} \right)}^{2}+4{z}_{0}a| V{| }^{2}}\right].$$

These bands are shown, for different temperatures, in Fig. 3a–c. Due to the hybridization of both bands, the quasiparticle weights in the lower (1) and upper (2) bands become momentum-dependent:

5 $${z}_{1,2\mathbf{p}}=\frac{(1+{z}_{0}a)({\varepsilon }_{1,2\mathbf{p}}-{z}_{0}{{\Delta }})-{z}_{0}a({\varepsilon }_{\mathbf{p}}^{(0)}-{z}_{0}{{\Delta }})}{2({\varepsilon }_{1,2\mathbf{p}}-{z}_{0}{{\Delta }})-({\varepsilon }_{\mathbf{p}}^{(0)}-{z}_{0}{{\Delta }})},$$

as shown in Fig. 3d–f. In standard Fermi liquid theory, the spectral weight is given by the residue of the respective Green’s function pole. Inserting these expressions into equation (1) leads to the curves shown in Figs. 2f,l and 3g,h. Here, the spectral distribution of the incident pulse, *W*(*ω*) in equation (1), is known from the experiment, and *α* and *T*_K_/*D* are the only adjustable parameters of this theory, where *D* is the free-electron conduction bandwidth. Symmetry implies that this result for the THz absorption is the same for particle-like (*Δ* > 0) and hole-like (*Δ* < 0) HF systems.

## Online content

Any methods, additional references, Nature Portfolio reporting summaries, source data, extended data, supplementary information, acknowledgements, peer review information; details of author contributions and competing interests; and statements of data and code availability are available at 10.1038/s41567-023-02156-7.

### Supplementary information

Supplementary Information Supplemental material.

Supplementary Data Fig. 1 Source data for Supplementary Fig. 1.

Supplementary Data Fig. 2 Source data for Supplementary Fig. 2.

Supplementary Data Fig. 3 Source data for Supplementary Fig. 3.

### Source data

Source Data Fig. 1 Source data for Fig. 1.

Image Source Fig. 1 Image source file for Fig. 1.

Source Data Fig. 2 Source data for Fig. 2.

Source Data Fig. 3 Source data for Fig. 3.

Source Data Fig. 4 Source data for Fig. 4.

Image Source Fig. 4 Image source file for Fig. 4.

## Data Availability

The datasets analysed in the current study are attached. Source data are provided with this paper. Any additional data are available from the corresponding authors upon request.

## Code availability

The codes associated with the numerical simulation of the band structure and the spectral weights that support this study are available from the corresponding authors upon request.
