# Supplementary material for: Critical slowing down near a magnetic quantum phase transition with fermionic breakdown
Source: Nat Phys. 2023 Jul 31;19(11):1605–10. doi: 10.1038/s41567-023-02156-7 (PMC10635820; doi:10.1038/s41567-023-02156-7)
Supplement: Supplementary file 1 — Supplemental material. [file 41567_2023_2156_MOESM1_ESM.pdf]

nature physics

Article

https://doi.org/10.1038/s41567-023-02156-7

Critical slowing down near a magnetic quantum phase transition with fermionic breakdown

In the format provided by the authors and unedited

Supplemental Material: Critical slowing down of fermions near a magnetic quantum phase transition

Chia-Jung Yang, Kristin Kliemt, Cornelius Krellner, Johann Kroha, Manfred Fiebig, and Shovon Pal
(Dated: July 13, 2023)

This supplement contains further information on how the THz absorption is extracted from the delayed response in the time trace, on the choice of the time window for extracting the THz absorption from the magnetic field- and temperature-dependent measurements, and on the low-temperature electrical resistivity of $YbRh_2Si_2$.

I. Extracting the spectral weight from the delayed pulse 1
II. Determining the time window 1
III. Temperature range for critical exponent 2
IV. Low-temperature resistivity 3

I. EXTRACTING THE SPECTRAL WEIGHT FROM THE DELAYED PULSE

The procedure for evaluating the spectral weight of the delayed response has been described in an elaborate manner for the case of the prototypical heavy-fermion compound $CeCu_{6\text{-x}}Au_x$ [1–3]. This spectral weight is intimately related to the THz absorption strength, see Eq. (1) of the main article. In the case of $CeCu_{6\text{-x}}Au_x$, the delayed response appears at around 6 ps, corresponding to the Kondo temperature of 8 K, and is well separated from the instantaneous response. In the present case, however, the Kondo temperature of $YbRh_2Si_2$ (YRS) is 25 K [4–8], which implies that the separation of the delayed response is around 1.95 ps. As a result, proper care has been taken in the data analysis to track the behavior of the THz absorption strength both as a function of temperature and external magnetic field.

In general, the steps for our data analysis include normalization, integration, and background subtraction. At first, all temperature-dependent time traces for fixed external magnetic fields are normalized in order to scale all traces to an overall identical power, i.e., the intensity integrated over the entire time trace equals one. In other words, the total reflectivity does not change with temperature. Figure S1(a) shows the THz time traces of the reference mirror and YRS at an external magnetic field of 70 mT for several temperatures. The THz absorption is then evaluated by integrating the squared electric field of these normalized traces over the time window 1.3 ps to 2.6 ps, indicated as the shaded area in Fig. S1(b). The justification for the choice of this interval is provided in the next section. To exclude the incoherent background [1], the spectral weight obtained as an offset at 300 K is subtracted from each temperature point.

II. DETERMINING THE TIME WINDOW

The THz electric-field envelope $\overline{E}(t)$ of the delayed response is proportional to the temporal change of occupation of photo-excited electrons. This takes the form [1]:

$$\overline{E}(t) = \frac{\overline{E}_0}{\cosh^2[2\pi(t/\tau_K - 1)]},$$

where $\overline{E}_0$ is the maximum pulse amplitude, and $\tau_K = h/k_B T_K$ is the coherence time corresponding to the Kondo temperature T_K. A schematic of the associated envelope is shown in Fig. S2(a). The time window over which the delayed pulse is integrated must encompass the essential part of the delayed pulse centered about τ_K which is set to ≈ 1.95 ps by the Kondo temperature of 25 K for YRS [4–8]. Let t_1 and t_2 be the lower and upper boundaries of this time interval, respectively. First, t_1 must be chosen to capture the maximum possible range of the delayed response pulse while excluding the tail of the instantaneous pulse. From Fig. S1(a), we see that the incident response (i.e., the response of the Pt-mirror) has a tail that extends until 1.2 ps. Upon integration of the reference response by varying the

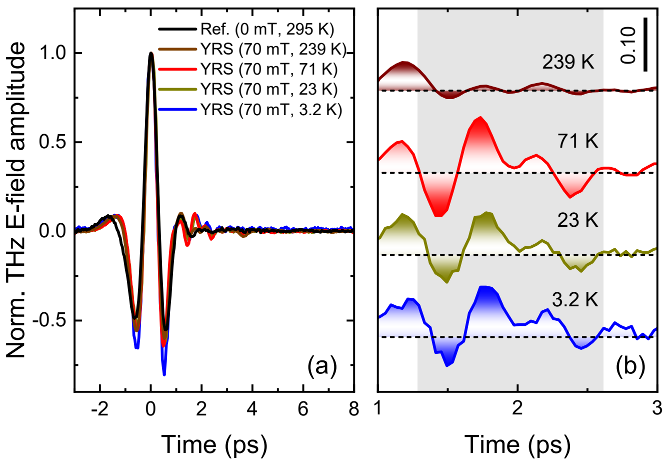

Figure S1. (a) The normalized temperature-dependent THz time traces from the reference platinum mirror at 0 mT, and the YRS sample at 70 mT. (b) Signal of the delayed response in YRS at 70 mT around 1.3 ps – 2.6 ps (shaded area). Note that the signals are plotted with offsets. The black-dashed lines indicate the zero THz electric field.

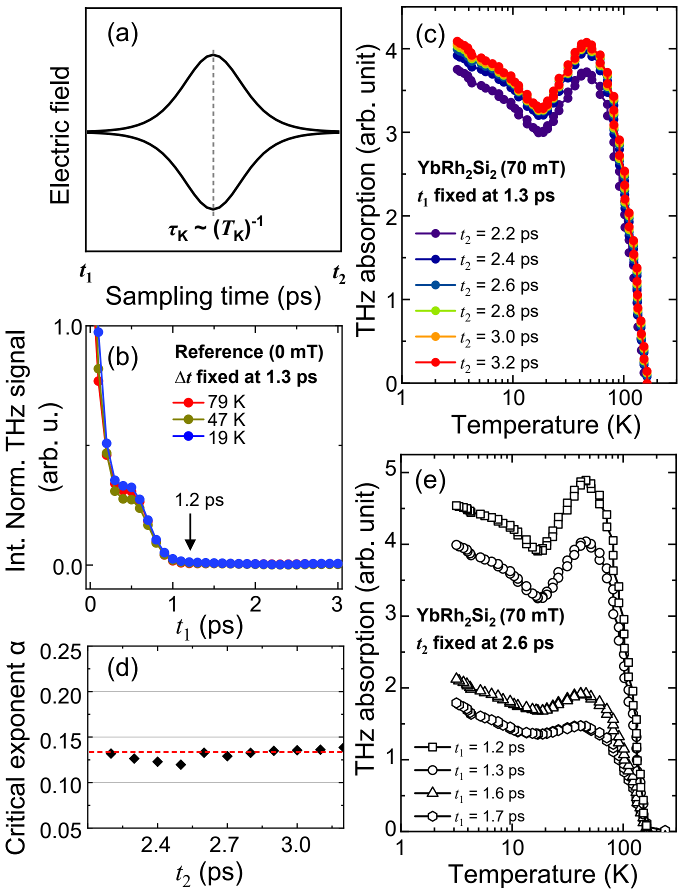

Figure S2. (a) Schematic of the envelope function. τ_K is the centre of the envelope, t_1 and t_2 are the lower and higher end of the envelope, respectively. (b) The integrated normalized THz signals obtained from temporal integration of the Pt reference response at various temperatures. (c) Temperature evolution of the THz absorption strength determined by varying t_2, keeping t_1 fixed at 1.3 ps. (d) The critical exponents evaluated from the corresponding curves in (b). The red-dashed line indicates $\alpha = 0.13$. (e) Temperature evolution of the THz absorption strength determined by varying t_1, keeping t_2 fixed at 2.6 ps.

position of the integration window (keeping the width of the window fixed at $\Delta t = 1.3$ ps), we observe that the reflected THz signals from the reference mirror reaches to zero when $t_1 = 1.2$ ps, see Fig. S2(b). Note that this response is temperature independent as portrayed in Fig. S2(b). Adding a buffer of another 0.1 ps, we set the t_1 value of our window to be 1.3 ps. Taking the time window centered symmetrically about $\tau_K = 1.95$ ps, this dictates the value of its upper boundary, $t_2 = 2.6$ ps. We now analyse the influence of variations of t_1 and t_2 on the measured results.

First, we vary t_2 from 2.2 ps to 3.2 ps, keeping t_1 fixed at 1.3 ps. The corresponding THz absorption traces are plotted in Fig. S2(c). We see that all curves coincide, with only a small reduction in overall magnitude for $t_2 = 2.2$ ps, since we start to exclude a certain portion of the delayed response from the long-time side. Note that in all cases the value of the critical exponent α (shown in Fig. S2(d)) remains the same within the tolerance value stated in the main manuscript.

Next, we explore the influence of varying t_1, keeping t_2 fixed at 2.6 ps. Being limited by the lower-bound value of t_1, we vary it from 1.2 ps to 1.7 ps. The corresponding THz absorption traces are plotted in Fig. S2(e). We see that for $t_1 < 1.3$ ps the low-temperature logarithmic increase of the measured THz absorption flattens out due to the inclusion of the instantaneous, temperature-independent response. On the other hand, for $t_1 > 1.3$ ps, we begin to exclude certain fractions of the delayed signal which leads to an overall decrease of the measured THz absorption signal and which, therefore, also affects the high-temperature logarithmic increase. Consequently, both scenarios lead to significant variations of the value of α. Importantly, however, the low-temperature logarithmic slope remains unchanged as t_1 is *increased* beyond 1.3 ps, and the high-temperature logarithmic slope remains constant as t_1 is *decreased* below 1.3 ps (see Fig. S2(e)). Having thus identified the origin of the variations of the logarithmic slopes at high and low temperatures, respectively, this means that $t_1 = 1.3$ ps is the optimal lower boundary of the time window which leads to a stable value of the critical exponent α. It sets the center of the time window to 1.95 ps and its full width to 1.3 ps.

At any quantum phase transition, the critical fluctuations extend in temperature up to the nearest intrinsic energy scale, beyond which non-universal behavior sets in. In the present case of a heavy-fermion system, this intrinsic scale is the Kondo temperature T_K, which in YRS is about 25 K. Hence, below this temperature quantum critical behavior is expected to be observed, and indeed, the logarithmic behavior near the critical B field is observed at and below about 10 K. Above T_K there is also a *non-universal* temperature dependence until the Kondo signal vanishes near 100 K. This is referred to as the "high-temperature Kondo scale"[7]. Note that our experiments at 2 K and above measure directly the order parameter, while the phase diagram below 2 K has been extracted previously from thermodynamic measurements, in particular, conductivity which shows non-Fermi liquid behavior[7]. It is, therefore, not surprising these different quantities show different behavior. However, it is important that, indeed, both types of measurements show non-Fermi liquid behavior as expected in the respective temperature regions. Our results are, therefore, completely consistent with the known low-temperature phase diagram and the energy scales in YRS.

III. TEMPERATURE RANGE FOR THE CRITICAL EXPONENT

The critical exponent is extracted by comparing the slopes of the logarithmic temperature behavior of $P(T)$ above T_K and in the low-temperature region, $\alpha = s_{low}/s_{high}$, see the main article. As we explain in the following, it is particularly important to locate the appropriate

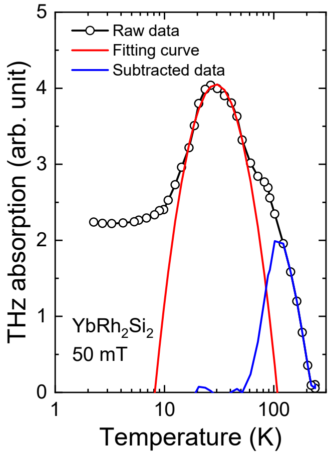

Figure S3. Temperature dependent THz absorption in YRS at an external magnetic field of 50 mT. The red solid curve represents the second-order parabolic fit around the main peak of the THz absorption. The blue solid curve is the THz absorption with the red curve subtracted. It represents the signal from the excited CEF states.

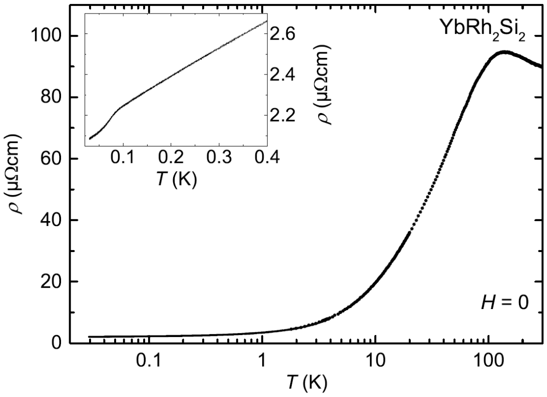

Figure S4. Temperature-dependent electrical resistivity of YRS in the absence of external magnetic field. The inset shows the resistivity kink at the Néel temperature [9].

temperature range for performing the logarithmic fitting to extract the high-temperature slope for the critical exponent.

All the temperature scans in Fig. 2 of the main article exhibit a kink at and above 100 K. This is identified with the first crystal-electric-field (CEF) excitation of YRS which is reported from neutron scattering and scanning tunneling microscopy measurements [4, 6]. The position of the CEF signal in our measurements can be estimated by fitting a second-order parabolic function to the peak of the THz absorption $P(T)$ and subtracting it from the temperature-dependent data, as shown in Fig. S3. Note that this position is somewhat below the literature value of the CEF excitation energy, because in our temperature-dependent measurements thermal occupation of the CEF excitation sets in already at lower temperatures than its excitation energy.

Therefore, we perform the fitting of the high-temperature logarithmic slope s_{high} in the temperature range below the kink and above the global maximum of the absorption strength, as indicated by the red, straight lines in Fig. 2 of the main article.

IV. LOW-TEMPERATURE RESISTIVITY

Temperature-dependent electrical resistivity measurements were carried out down to 30 mK, as shown in Fig. S4. The inset in Fig. S4 shows the anomaly at the Néel temperature, $T_{\mathrm{N}} = 70\,\mathrm{mK}$. We can also see the Kondo maximum in the logarithmic plot at around 100 K. In addition, we find a high residual resistivity ratio of $\rho_{300\,\mathrm{K}}/\rho_{0.03\,\mathrm{K}} = 43$, where $\rho_{300\,\mathrm{K}}$ and $\rho_{0.03\,\mathrm{K}}$ are the resistivity of YRS at 300 K and 30 mK, respectively. This high value indicates that the samples under investigation are of good quality with only a small impurity or defect concentration.

The resistivity measurements from 300 K to 1.8 K were performed in a commercial Physical Property Measurement System from Quantum Design. The resistivity was obtained with the AC-transport option in a four-point geometry. The contacts were manually prepared on the surface of the crystals with silver epoxy and 25 μm thick platinum wire [10]. On the other hand, the resistivity measurements down to 30 mK were performed in a 3He-4He dilution refrigerator from Oxford Instruments using a standard four-terminal AC technique, similar to the resistivity measurements described in Ref. [9, 11].

[1] C. Wetli, S. Pal, J. Kroha, K. Kliemt, C. Krellner, O. Stockert, H. v. Löhneysen, and M. Fiebig, Nat. Phys. **14**, 1103 (2018).

[2] S. Pal, C. Wetli, F. Zamani, O. Stockert, H. v. Löhneysen, M. Fiebig, and J. Kroha, Phys. Rev. Lett. **122**, 096401 (2019).

[3] C.-J. Yang, S. Pal, F. Zamani, K. Kliemt, C. Krellner, O. Stockert, H. v. Löhneysen, J. Kroha, and M. Fiebig, Phys. Rev. Research **2**, 033296 (2020).

[4] O. Stockert, M. Koza, J. Ferstl, A. Murani, C. Geibel, and F. Steglich, Physica B: Condensed Matter **378-380**, 157 (2006).

[5] S. Kimura, J. Sichelschmidt, J. Ferstl, C. Krellner, C. Geibel, and F. Steglich, Phys. Rev. B **74**, 132408 (2006).

[6] S. Ernst, S. Kirchner, C. Krellner, C. Geibel, G. Zwicknagl, F. Steglich, and S. Wirth, Nature **474**, 362 (2011).

[7] S. Seiro, L. Jiao, S. Kirchner, S. Hartmann, S. Friedemann, C. Krellner, C. Geibel, Q. Si, F. Steglich, and S. Wirth, Nature Communications **9**, 3324 (2018).

[8] L. Prochaska, X. Li, D. C. MacFarland, A. M. Andrews, M. Bonta, E. F. Bianco, S. Yazdi, W. Schrenk, H. Detz, A. Limbeck, Q. Si, E. Ringe, G. Strasser, J. Kono, and S. Paschen, Science **367**, 285 (2020).

[9] S. Hamann, Ph.D. Thesis, Quantenmultikritikalität in $YbRh_2Si_2$, TU Dresden (2018).

[10] K. Kliemt, M. Peters, F. Feldmann, A. Kraiker, D.-M. Tran, S. Rongstock, J. Hellwig, S. Witt, M. Bolte, and C. Krellner, Crystal Research and Technology **55**, 1900116 (2020).
[11] S. Hamann, J. Zhang, D. Jang, A. Hannaske, L. Steinke, S. Lausberg, L. Pedrero, C. Klingner, M. Baenitz, F. Steglich, C. Krellner, C. Geibel, and M. Brando, Phys. Rev. Lett. **122**, 077202 (2019).
