# Supplementary figures and images for: Critical slowing down near a magnetic quantum phase transition with fermionic breakdown
Source: Nat Phys. 2023 Jul 31;19(11):1605–10. doi: 10.1038/s41567-023-02156-7 (PMC10635820; doi:10.1038/s41567-023-02156-7)

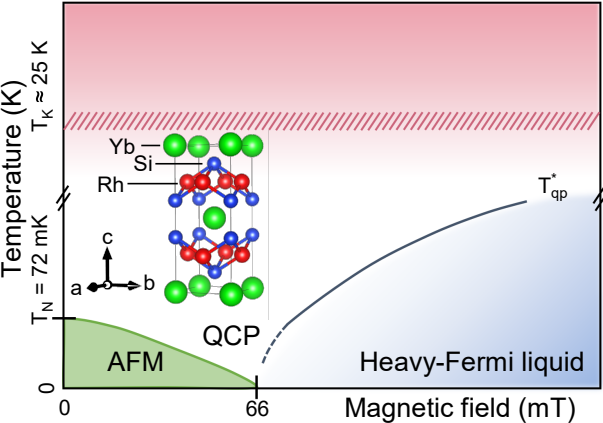

Supplement: Supplementary file 6 — Image source file for Fig. 1. [file 41567_2023_2156_MOESM6_ESM.pdf]

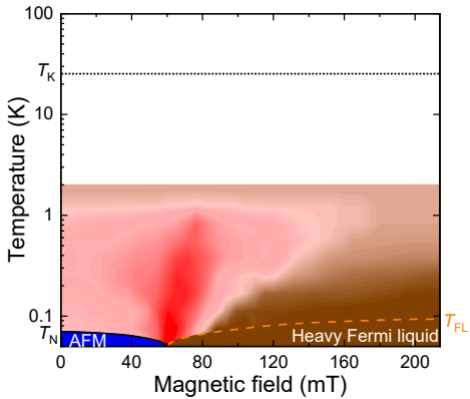

Supplement: Supplementary file 10 — Image source file for Fig. 4. [file 41567_2023_2156_MOESM10_ESM.pdf]
